# Study of the Role of Cytosolic Phospholipase A_2_ Alpha in Eicosanoid Generation and Thymocyte Maturation in the Thymus

**DOI:** 10.1371/journal.pone.0126204

**Published:** 2015-05-13

**Authors:** Matthieu Rousseau, Gajendra S. Naika, Jean Perron, Frederic Jacques, Michael H. Gelb, Eric Boilard

**Affiliations:** 1 Centre de Recherche en Rhumatologie et Immunologie, Centre de Recherche du Centre Hospitalier Universitaire de Québec, Faculté de Médecine de l’Université Laval, Québec, QC, Canada; 2 Department of Chemistry, University of Washington, Seattle, WA, the United States of America; 3 Centre de Recherche du Centre Hospitalier Universitaire de Québec, Faculté de Médecine de l’Université Laval, Québec, QC, Canada; Universidade Federal do Rio de Janeiro, BRAZIL

## Abstract

The thymus is a primary lymphoid organ, home of maturation and selection of thymocytes for generation of functional T-cells. Multiple factors are involved throughout the different stages of the maturation process to tightly regulate T-cell production. The metabolism of arachidonic acid by cyclooxygenases, lipoxygenases and specific isomerases generates eicosanoids, lipid mediators capable of triggering cellular responses. In this study, we determined the profile of expression of the eicosanoids present in the mouse thymus at different stages of thymocyte development. As the group IVA cytosolic phospholipase A_2_ (cPLA_2_α) catalyzes the hydrolysis of phospholipids, thereby generating arachidonic acid, we further verified its contribution by including cPLA_2_α deficient mice to our investigations. We found that a vast array of eicosanoids is expressed in the thymus, which expression is substantially modulated through thymocyte development. The cPLA_2_α was dispensable in the generation of most eicosanoids in the thymus and consistently, the ablation of the cPLA_2_α gene in mouse thymus and the culture of thymuses from human newborns in presence of the cPLA_2_α inhibitor pyrrophenone did not impact thymocyte maturation. This study provides information on the eicosanoid repertoire present during thymocyte development and suggests that thymocyte maturation can occur independently of cPLA_2_α.

## Introduction

The thymus has a central role in the immune system as it supports the development, the differentiation and the selection of T-cells [1–3]. Thymic development of the T-cell precursors is finely regulated. Firstly, the T-cell precursors originating from the bone marrow enter in the thymus through the cortex. These immature T-cells, called thymocytes, differently express the T-cell receptor (TCR) co-receptors CD4 and CD8 at their surface, an indication of the T-cell maturation state. Owing to the lack of expression of CD4 and CD8 immediately after their entrance in the cortex, the most immature T-cells are identified as double negative (DN) thymocytes (CD4^-^/CD8^-^). Secondly, after a productive rearrangement of the TCR β locus and expression of pre-TCR, thymocytes initiate the expression of CD4 and CD8 and are recognized as double positive (DP) thymocytes. Finally, the DP thymocytes undergo positive and negative selections driven by dendritic cells, cortical and medullar thymic epithelial cells. These two selection processes eliminate by apoptosis the thymocytes considered as useless and self-reactive. The positively selected thymocytes then migrate to the medulla and egress from the thymus as single positive (SP) thymocytes (*i*.*e*. CD4^+^/CD8^-^ and CD4^-^/CD8^+^).

Multiple factors tightly regulate the formation of T-cells throughout the different stages of the maturation process. Cytokines and chemokines for instance are involved in thymocyte survival, differentiation, selection and guidance through the thymus [3, 4]. Eicosanoids are lipid mediators derived from fatty acids, such as arachidonic acid (AA), and are synthesized primarily by three classes of enzymes, cyclooxygenases 1 (COX-1) or 2 (COX-2), lipoxygenases (LOX) and cytochrome P450 mono-oxygenases. The role of eicosanoids in regulation of immunity is well documented. For example, prostaglandin E_2_ (PGE_2_), prostanoid formed via the concerted action of cyclooxygenases (COX-1 and COX-2) and PGE synthase, induces Th2 polarization by modulating cytokine production of antigen presenting cells and T-cells [5]. PGE_2_ inhibits the production of interleukin (IL)-2, IL-12 and interferon-γ by monocytes, T-cells and antigen presenting cells, it decreases the responsiveness of IL-12 receptors by peripheral blood mononuclear cells and T-cells and increases T-cell production of IL-4, IL-5, and IL-10 [5–9]. Alternatively, PGE_2_ has also a role in the differentiation of Th17-cells, and nanomolar concentrations of this eicosanoid suffice to promote Th1 differentiation, whereas higher concentrations of PGE_2_ suppress this process [10–13]. Furthermore, it was shown that PGE_2_ suppresses allergic reactions through the PGE_2_ receptor 3 (EP3) [14] and promotes induction of FOXP3^+^ CD4^+^ CD25^+^ adaptive regulatory T-cells that regulate immune responses [15–17]. Finally, PGE_2_ supports the maturation of B-lymphocytes into IgE-producing plasma cells [18, 19]. While the enzymatic machinery necessary for eicosanoid biosynthesis (COX-1 and COX-2, prostaglandin synthases, thromboxane synthase, 5LOX, 15LOX, P450 mono-oxygenase) and the eicosanoid receptors (PGE_2_ receptors (EP), thromboxane receptor, leukotriene B4 receptors (BLT1 and BLT2)) are expressed in the thymus [20–32], little is known regarding the eicosanoids present in the thymus through different stages of thymocyte maturation.

More than 90% of the thymocytes retrieved in the thymus are synchronized as DN on day 15.5 of the mouse embryonic development (E15.5). Thymocyte maturation then progresses, and 70–80% of the thymocytes examined on embryonic day 18.5 are then DP. Fetal thymic organ cultures (FTOC) are therefore frequently utilized to study the impact of gene ablation or protein inhibition on thymocyte development [33, 34]. FTOCs were previously used to assess the contribution of prostanoids in the thymus [21, 35]. In a first study, which included fetal thymuses isolated from COX-1 and COX-2 knockout (KO) mice and inhibitors of COX-1 and COX-2, COX-1-dependent PGE_2_ production was shown involved in the transition from DN to DP T-cells whereas the COX-2-dependent PGE_2_ production was shown necessary in generation of CD4^+^ SP T-cells. Furthermore, using specific agonists of prostanoid receptors, it was confirmed that these effects were mediated through activation of the PGE_2_ receptors EP-2 and EP-1. Taken together, these observations point to an important role of AA metabolites, most specifically PGE_2_, in T-cell development in the thymus. However, a second study showed that the maturation of thymocytes remained intact in culture of fetal thymuses isolated from mice deficient in COX-1, COX-2, EP-1, EP-2 and mice deficient for both COX-1 and COX-2 [35]. While the addition of a COX-2 inhibitor (NS-398) to thymic cultures reduced the formation of CD4^+^ T-cells, this effect was unspecific as it was also present in FTOCs from COX-2 deficient mice and it was not reversed by the exogenous addition of PGE_2_ (up to 10μM) [35]. Thus, whether prostanoids actually participate in thymocyte development remains unclear.

AA, which is mainly esterified at the *sn*-2 position of phospholipids, has to be released from the membrane phospholipids to be metabolized into eicosanoids. The availability of AA is thus a rate-limiting step for the production of eicosanoids [36]. Phospholipases A_2_ (PLA_2_) catalyze the hydrolysis of phospholipids in *sn*-2, generating free fatty acids and lysophospholipids [37]. So far, more than 20 mammalians PLA_2_s have been described. The PLA_2_ repertoire includes; 1) the secreted PLA_2_s (dependent of calcium), 2) the intracellular PLA_2_s of group VI independent of calcium, 3) the intracellular PLA_2_s of group IV dependent of calcium (with the exception of the group IVC (cPLA_2_ gamma), which does not rely on calcium for its activity), 4) the lysosomal PLA_2_, 5) the adipose-specific PLA_2_ and 6) the platelet-activating factor acetylhydrolases. The most studied and best-described PLA_2_ is the cytosolic PLA_2_ of group IVA, also called cPLA_2_α. This enzyme is ubiquitously expressed in mammalian cells, and cPLA_2_α gene ablation in mice showed its critical role in fertility, particularly in fetus implantation and labor [38, 39]. Importantly, the exogenous injection of PGE_2_ and of a stable analog of PGI_2_ (carbaprostacyclin) restored normal implantation in cPLA_2_α deficient mice [40], further supporting the notion of functional coupling between cPLA_2_α and prostaglandins. In concurrent studies, the function of cPLA_2_α in eicosanoid production in a context of inflammation is also exemplified, as cPLA_2_α deficient mice were resistant to experimental asthma, and the macrophages isolated from these mice failed to produce PGE_2_, platelet activating factor, leukotriene B4 and leukotriene C4 [38, 39]. A series of subsequent studies confirmed a dominant role of cPLA_2_α in eicosanoid production in several processes, including immunity, reproduction, inflammation and cancer [7, 37–45]. While cPLA_2_α is expressed in thymocytes [46], whether it plays a role in eicosanoid generation and thymocyte maturation is unknown.

For this study, we portrayed the eicosanoids produced in the thymus at different stages of thymocyte maturation and considered the potential role of cPLA_2_α in this process. As the role of eicosanoids in the thymus has been invoked, we further hypothesized that cPLA_2_α might contribute to thymocyte maturation. We found that the production of eicosanoids is modulated accordingly to the maturation of thymocytes, and that the production of eicosanoids and thymocytes can proceed independently of cPLA_2_α.

## Materials and Methods

### Ethic statement

This study was reviewed and approved by our institutional review board (Comité Éthique de la Recherche du CHU de Québec) before the study began.

Human thymuses from newborns and young children were obtained under an approved institutional review board protocol (Comité Éthique de la Recherche du CHU de Québec) following written consent of the parents after a cardiac surgery (CHU de Quebec). This consent procedure was approved by the Comité Éthique de la Recherche du CHU de Québec.

In this study, Guidelines of the Canadian Council on Animal Care were followed in a protocol approved by the Animal Welfare committee at Laval University (Quebec City, Canada) and all efforts were made to minimize suffering. Fetal thymus harvesting was performed after euthanasia of fetuses on ice. Adult thymuses were obtained after an isoflurane anesthesia followed by euthanasia with CO_2_.

### Mice and genotyping

C57BL/6J mice were obtained from The Jackson Laboratory. cPLA_2_α deficient mice [38] were backcrossed up to the tenth generation in C57BL6/J background. The reproduction of cPLA_2_α deficient mice was maintained by crossing heterozygous males and females, and the littermate cPLA_2_α wild type (WT) and cPLA_2_α KO were used for our experiments. The identification of cPLA_2_α genotypes was performed using DNA isolated from mouse tail. The tails were digested with DirectPCR Lysis Reagent (Tail) (Viagen Biotech) and Proteinase K (Invitrogen) according to the manufacturer protocol and PCR amplification was performed using HotStarTaq DNA Polymerase (Qiagen) and the following primers: cPLA_2_ α forward (5′-TTCTCTGGTGTGATGAAGGC-3′), cPLA_2_ α reverse 5′-AAACTGACTGTAGCATCACAC-3′), NeoForward (cPLA_2_α KO) (5′- ATCGCCTTCTTGACGAGTTC-3′). The following PCR steps were used: 15 minutes at 95°c, 35 cycles of 45 seconds at 94°c, 60 seconds at 65°c and 60 seconds at 72°c and the final step is 10 minutes at 72°c. The PCR products were then separated on 1.5% agarose gel containing ethidium bromide. The WT and KO products were distinguished by visualization of bands at 224 and 570 bp, respectively.

### Fetal Thymic Organ Culture

FTOCs were produced as previously described [33, 34]. In brief, fecundation was timed from the first day of plug observation (day 0.5). Mouse fetuses were harvested from timed pregnant mice on gestational day 15.5. The fetal thymuses were cultured on 0.8μm Isopore Membrane filters (Millipores) placed on the surface of 12 well plates containing RPMI 1640 (Wisent) supplemented with 10% FBS for mouse myeloid colony forming cells (Stemcell Technologies), 1% penicillin/streptomycin (Wisent), 1% L-glutamine (Wisent) and 1% of 2-mercaptoethanol (Gibco). FTOC were fed daily by complete medium replacement with solvent control (DMSO or ethanol) or the following compounds: cPLA_2_α inhibitor pyrrophenone (Cayman Chemical), arachidonic acid (Nu Chek Prep) and prostaglandin E_2_ (Cayman Chemical). Fetal thymuses were cultured for 5 days at 37°C with 5% CO_2_.

### Human thymus

Small sections of human thymuses (≈ 2mm^3^) were cultured as already described for mouse FTOCs. The human FTOCs-like were fed daily by complete medium replacement with solvent control (DMSO) or pyrrophenone (Cayman Chemical) for 5 days at 37°C with 5% CO_2_.

### Flow cytometry analysis

Thymuses were mechanically dissociated into single cell suspensions in PBS. The absolute cell number present in each thymus was determined by cell counting and labeling with fluorochrome-conjugated antibodies was performed according to the manufacturer protocols. The following antibodies were used: PE-Cy7 Hamster Anti-Mouse CD3e (145-2C11), PE Rat Anti-Mouse CD4 (RM4-5), APC Rat Anti-mouse CD8a (53–6.7), PE-Cy7 mouse Anti-Human CD3 (clone SK7), PE mouse Anti-human CD4 (RPA-T4) and APC mouse Anti-Human CD8 (RPA-T8). All antibodies and their related isotype controls were purchased from BD Biosciences. Flow cytometry analysis was performed on a BD FACSCanto II Flow cytometer (BD Biosciences, San Jose, California, USA) and analyzed using FlowJo software (Ashland, Oregon USA).

### Mass spectrometry analysis of eicosanoids

Eicosanoids from 1ml FTOC supernatants and crushed mouse adult thymuses were analyzed by combined liquid chromatography/tandem mass spectrometry, as already described [47]. The FTOC supernatants were collected daily and conserved at -80°C before analysis. Thymuses from cPLA_2_α WT and KO adult mice were crushed in 1ml PBS 1X and conserved at -80°C before analysis. Culture media (in absence of FTOC) was used as negative control for our analyses. Deuterium standards purchased from Cayman Chemical were used to detect the set of eicosanoids listed in the Table 1.

**Table 1:** Set of eicosanoids evaluated in FTOCs and adult mouse thymuses.

Eicosanoids	Detection	
	FTOC	Adult thymus
LTB_4_ products	Detected	Detected
LTC_4_	Not detected	Not detected
LTD_4_	Not detected	Not detected
LTE_4_	Not detected	Not detected
5-HETE	Not detected	Detected
8-HETE	Not detected	Not detected
11-HETE	Detected	Detected
12-HETE	Not detected	Detected
Tetranor-12-HETE	Not detected	Not detected
15-HETE	Not detected	Detected
5-OxoETE	Detected	Detected
15-OxoETE	Not Detected	Detected
5,6-DiHETE	Detected	Detected
5,15-DiHETE	Detected	Detected
Resolvin D1	Detected	Detected
Resolvin D2	Not detected	Not detected
Resolvin E1	Not detected	Not detected
5,6-LXA_4_	Detected	Detected
5,14-LXB_4_	Detected	Detected
PGD_2_	Not detected	Detected
PGE_2_	Detected	Detected
PGF_2_α	Detected	Detected
11β-PGF_2_α	Not detected	Not detected
2,3-Dinor-11β-PGF_2_α	Not detected	Not detected
6-Keto PGF_1_α	Detected	Detected
2,3-Dinor-6-Keto PGF_1_α	Detected	Detected
TXB_2_	Detected	Detected
2,3-Dinor TXB_2_	Detected	Detected
11-dehydro TXB_2_	Not detected	Not detected
12-HHTrE	Detected	Detected
8,9-DHET	Detected	Detected
11,12-DHET	Not detected	Not detected
14,15-DHET	Detected	Detected

Eicosanoids* from FTOC supernatants and adult mouse thymuses were measured by combined liquid chromatography/tandem mass spectrometry.

* Leukotriene (LT); Hydroxyeicosatetraenoic acid (HETE); Oxo-eicosatetraenoic acid (OxoETE); Dihydroxy-eicosatetraenoic acid (DiHETE); Lipoxin (LX); Prostaglandin (PG); Thromboxane (TXB); Hydroxy-heptadecatrienoic acid (HHTrE); Dihydroxy-eicosatrienoic acid (DHET).

### RT-QPCR

Total RNA was extracted from C57BL6/J mouse thymus using TRIzol reagent (Invitrogen). All RNA samples were treated with DNase I to eliminate residual genomic DNA prior to amplification. cDNA was synthesized using MLV-RT (invitrogen), real time quantitative PCR analysis was performed using a Rotorgene apparatus (Montreal Biotech, Canada) and levels of cPLA2α mRNA were determined using SYBR Green dye (Invitrogen) and the following primer pair: cPLA2 α forward (5′-CAGCTCTCAGGATTCCTTCGA-3′), cPLA2α reverse (5′- TCATATATTCGTTC AAATTCATCTGGAT -3′), ribo S15 foward (5′-ATGTCCT ATGAGCAACTGATGCA -3′), ribo S15 reverse (5′- GCCGAAGACCACGGTTCA-3′). The relative expression of the cPLA_2_α gene was determined using the 2^-ΔCt^ methods. In brief the ΔCt is cPLA_2_α Ct—ribo S15 Ct.

### Statistical analyses

All data are presented as mean ± SEM. Statistical significance between 2 groups was determined using unpaired Student *t* tests. All the statistical analyses were performed using Prism software 4.00 (GraphPad Software, CA, USA).

## Results

### Eicosanoid profiling during thymocyte maturation

**Fig 1:**
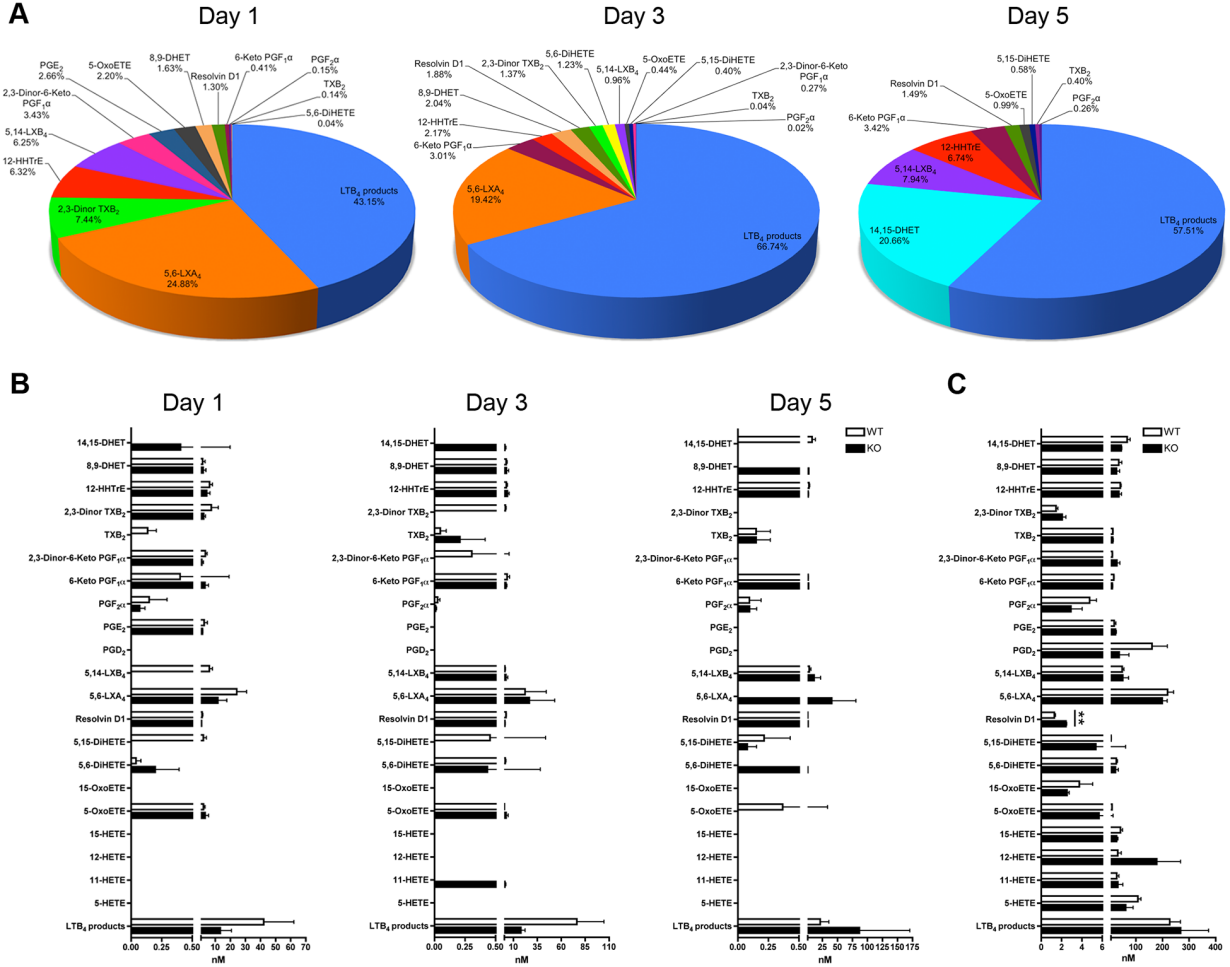
Eicosanoid profiles of cPLA_2_α WT and KO FTOC supernatants and adult mouse thymuses. **A.** Expression distribution of the eicosanoids present in cPLA_2_α WT FTOCs. The supernatants of FTOCs were collected at the indicated time of culture and the eicosanoid profiles were determined by combined liquid chromatography/tandem mass spectrometry. Data are mean of 3 different supernatants. **B.** Eicosanoid profiles of cPLA_2_α WT and KO FTOC supernatants. The supernatants of FTOCs were collected at the indicated time of culture and eicosanoid profiles were determined by combined liquid chromatography/tandem mass spectrometry. Data are mean ± SEM of 3 different supernatants. **C.** Eicosanoid profiles of cPLA_2_α WT and KO thymuses from adult mice. Adult thymuses were mechanically disrupted and eicosanoid profiles were determined by combined liquid chromatography/tandem mass spectrometry. ** P< .01, data are means ± SEM of 3 cPLA_2_α WT thymuses and 2 cPLA_2_α KO thymuses.

To determine the eicosanoids produced by the thymus through different stages of thymocyte maturation, we compared the lipid profile generated in FTOC supernatants (E15.5) after 1, 3 and 5 days of culture. The full-set of eicosanoids that was evaluated is presented in Table 1. LTC_4_, LTD_4_, LTE_4_, 8-HETE, Tetranor-12-HETE, Resolvin D2, Resolvin E1, 11α-PGF_2_α, 2,3-Dinor-11β-PGF_2_α, 11-dehydro TXB_2_ and 11,12-DHET were undetectable in FTOC supernatants and adult mouse thymuses. Furthermore, we found profound changes in the eicosanoid expression profile during the course of thymocyte maturation, with LTB_4_ and LXA_4_ representing the majority (>50%) of the eicosanoids expressed through the first 3 days of culture (Fig 1A and 1B, left and middle panel). At day 5 of culture, 14,15-DHET was the second most abundant lipid mediator produced by FTOCs after LTB_4_, while LXA_4_ appeared essentially absent (Fig 1A and 1B, right panel). Next, we wished to verify the expression of eicosanoids present in the thymus of adult mice (6–8 weeks). In this case, we found that LTB_4_ remains among the most abundant lipid mediator present in the thymus, followed by LXA_4_ and 5-HETE (Fig 1C).

The production of eicosanoids by FTOCs, adult thymus and the modulation of their production during the course of thymocyte development, prompted our examination of the role of cPLA_2_α. Using FTOCs and adult thymuses from cPLA_2_α deficient mice, we observed that the majority of the most abundant eicosanoids could be produced independently of the expression of cPLA_2_α (Fig 1B and 1C). The ablation of the gene coding for cPLA_2_α led to the absence of 5,15-DiHETE, 5,14-LXB_4_ and TXB_2_ at day 1, of 2,3-Dinor TXB_2_, 2,3-Dinor-6-Keto PGF_1_α and 5,15-DiHETE at day 3 and of 14,15-DHET and 5-OxoETE at day 5 of culture in FTOCs, suggesting that cPLA_2_α is implicated in the generation of these lipids (Fig 1B). Furthermore, 14,15-DHET at day 1, 14,15-DHET and 11-HETE at day 3, 8,9-DHET, 5,6-LXA_4_ and 5,6-DiHETE at day 5 were only detected in cPLA_2_α KO FTOC supernatants (Fig 1B) while significantly more Resolvin D1 was observed in absence of cPLA_2_α in mouse adult thymuses (Fig 1C), suggesting that cPLA_2_α expression can also negatively regulate the production of some eicosanoids. Taken together, these results demonstrate that the production of eicosanoids is modulated accordingly to the development stages of thymocytes, and that the majority of the eicosanoids detected in mouse fetal and adult thymuses are produced independently of cPLA_2_α. These observations also point to a contribution of cPLA_2_α in expression of a subset of less abundant eicosanoids in the thymus.

### The disruption of the cPLA_2_α gene does not affect the maturation of thymocytes in FTOC

**Fig 2:**
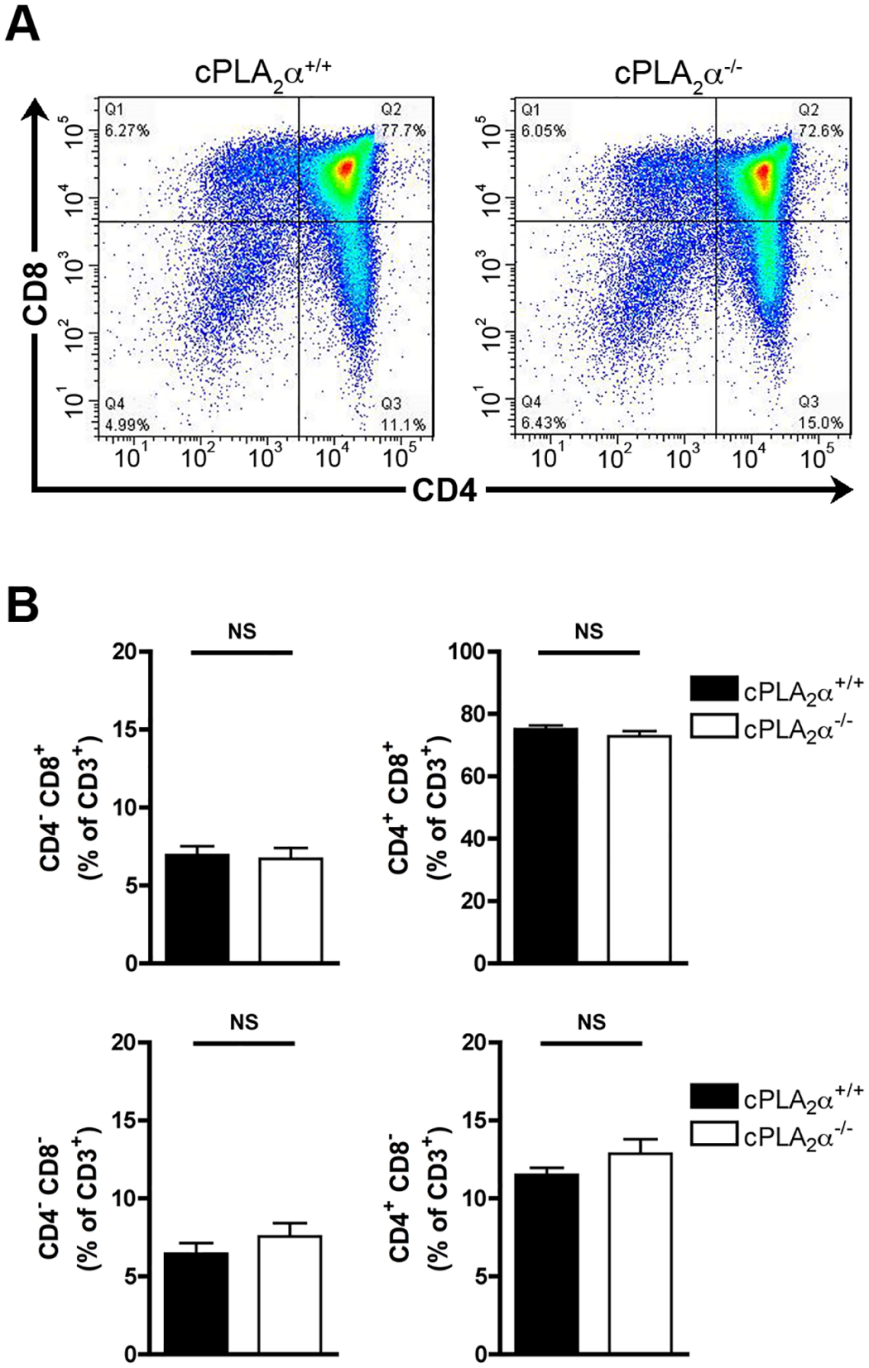
The disruption of the cPLA_2_α gene does not impact thymocyte maturation in FTOC. **A.** Representative thymocyte subpopulation distribution in WT and KO cPLA_2_α FTOC. After 5 days of culture, the identification of thymocytes with fluorochrome-conjugated antibodies directed against CD3, CD4 and CD8 was determined by flow cytometry. **B.** WT and KO cPLA_2_α fetal thymuses were cultured during 5 days as FTOCs. After mechanical dissociation of fetal thymuses, the thymocytes were labeled with fluorochrome-conjugated antibodies directed against CD3, CD4, and CD8, and analyzed by flow cytometry. Data are mean ± SEM of 6 independent experiments and the number of fetal thymuses for each genotype is: cPLA_2_^+/+^ (n = 17); cPLA_2_^-/-^ (n = 9). NS (non significant).

Although cPLA_2_α appeared dispensable for the biosynthesis of most eicosanoids, subtle changes in the lipid expression profile in the thymus were observed in absence of cPLA_2_α. Furthermore, cPLA_2_α might be implicated in the generation of eicosanoids in discrete cellular lineages in the thymus, which might not be possible to estimate when measuring the complete pool of eicosanoids produced by the entire organ. We thus wished to verify whether the cPLA_2_α is implicated in thymocyte maturation, and we firstly used a genetic approach in FTOCs [33, 34]. The thymocytes present in the cultured thymus from cPLA_2_α WT and KO littermate mice were examined cytofluorometrically, and no differences in their maturation were observed. Indeed, the four subpopulations studied, the DN (CD3^+^/CD4^-^/CD8^-^), the DP (CD3^+^/CD4^+^/CD8^+^) and the SP (CD3^+^/CD4^+^/CD8^-^ and CD3^+^/CD4^-^/CD8^+^) thymocytes showed the same repartition in the cPLA_2_α WT and KO FTOCs (Fig 2A and 2B). In light of these results, cPLA_2_α is dispensable for the maturation of thymocytes in mice.

### Evaluation of the impact of the cPLA_2_α inhibitor pyrrophenone on thymocyte maturation

**Fig 3:**
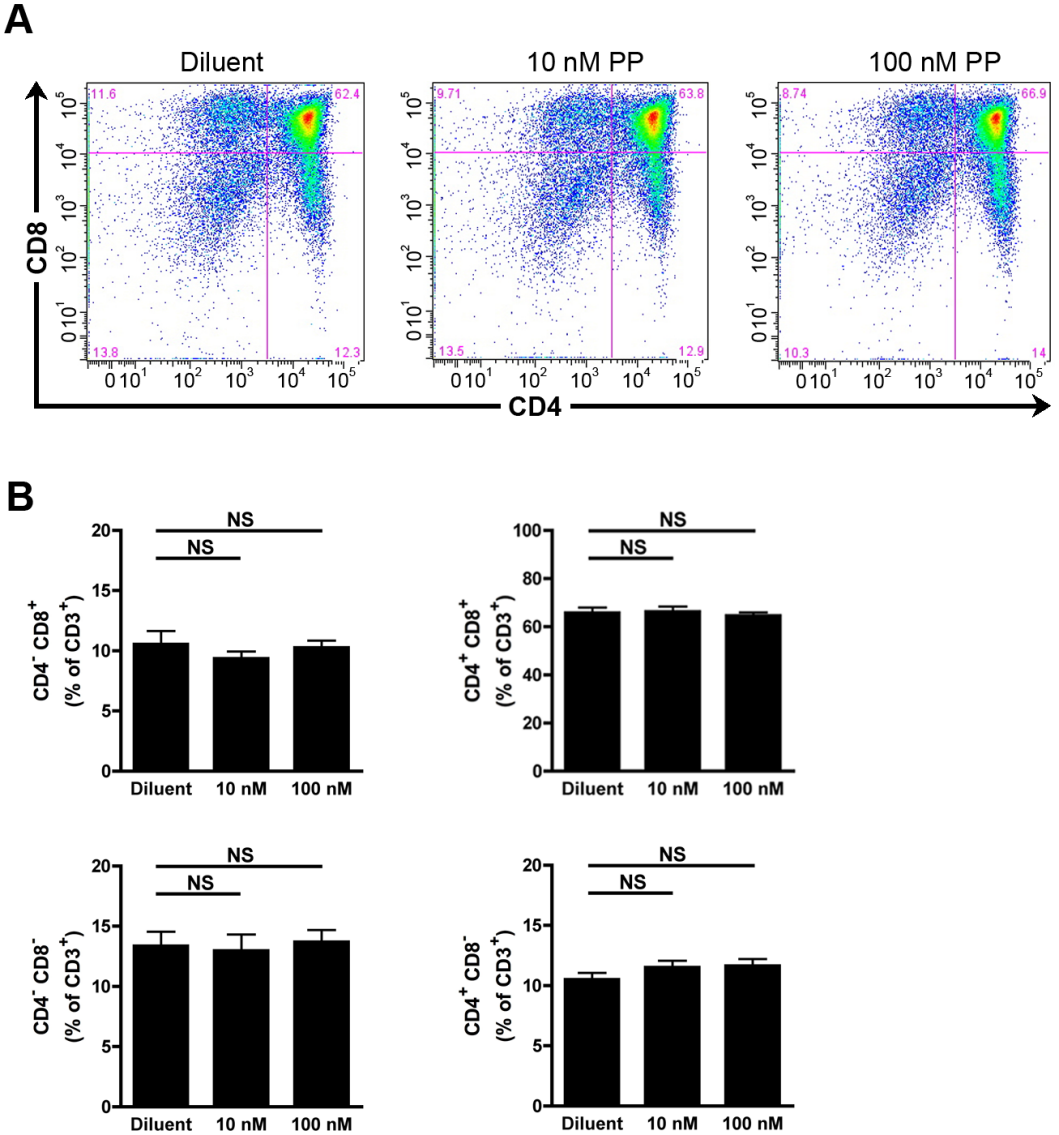
Pharmacological blockade of cPLA_2_α does not affect thymocyte maturation in FTOC. **A.** Representative thymocyte subpopulation distribution in WT cPLA_2_α FTOC after 5 days of culture in absence or presence of indicated concentrations of PP. Thymocytes were identified with fluorochrome-conjugated antibodies directed against CD3, CD4 and CD8 by flow cytometry. **B.** WT cPLA_2_α fetal thymuses were cultured during 5 days as FTOCs in absence or presence of indicated concentrations of PP. After mechanical dissociation of fetal thymuses, the thymocytes were labeled with fluorochrome-conjugated antibodies directed against CD3, CD4, and CD8 and analyzed by flow cytometry. Data are mean ± SEM of 5 independent experiments and the number of fetal thymuses for each condition is: Diluent (n = 10); 10nM PP (n = 8); 100nM PP (n = 12). NS (non significant).

**Fig 4:**
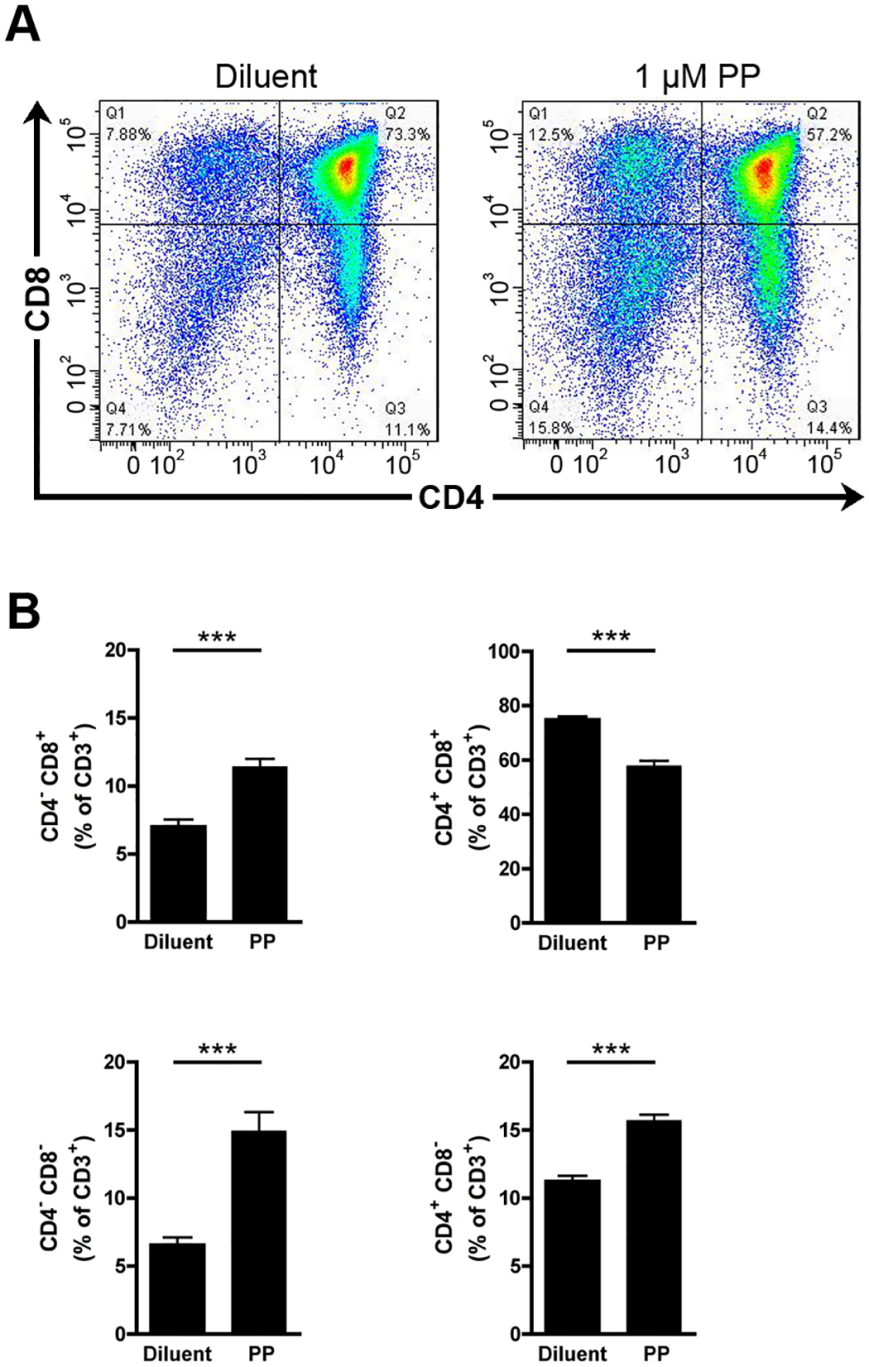
cPLA_2_α inhibition by high concentration of PP impacts thymocyte maturation in FTOC. **A.** Representative thymocyte subpopulation distribution in WT cPLA_2_α FTOC after 5 days of culture in absence or presence of 1μM of PP. Thymocytes were identified cytofluorometrically using fluorochrome-conjugated antibodies directed against CD3, CD4 and CD8. **B.** WT cPLA_2_α fetal thymuses were cultured during 5 days as FTOCs in absence or presence of 1μM of PP. After mechanical dissociation of fetal thymuses, the thymocytes were labeled with fluorochrome-conjugated antibodies directed against CD3, CD4, and CD8 and analyzed by flow cytometry. Data are mean ± SEM of 9 independent experiments and the number of fetal thymuses for each condition is: Diluent (n = 22); 1μM PP (n = 13). *** P< .001.

**Fig 5:**
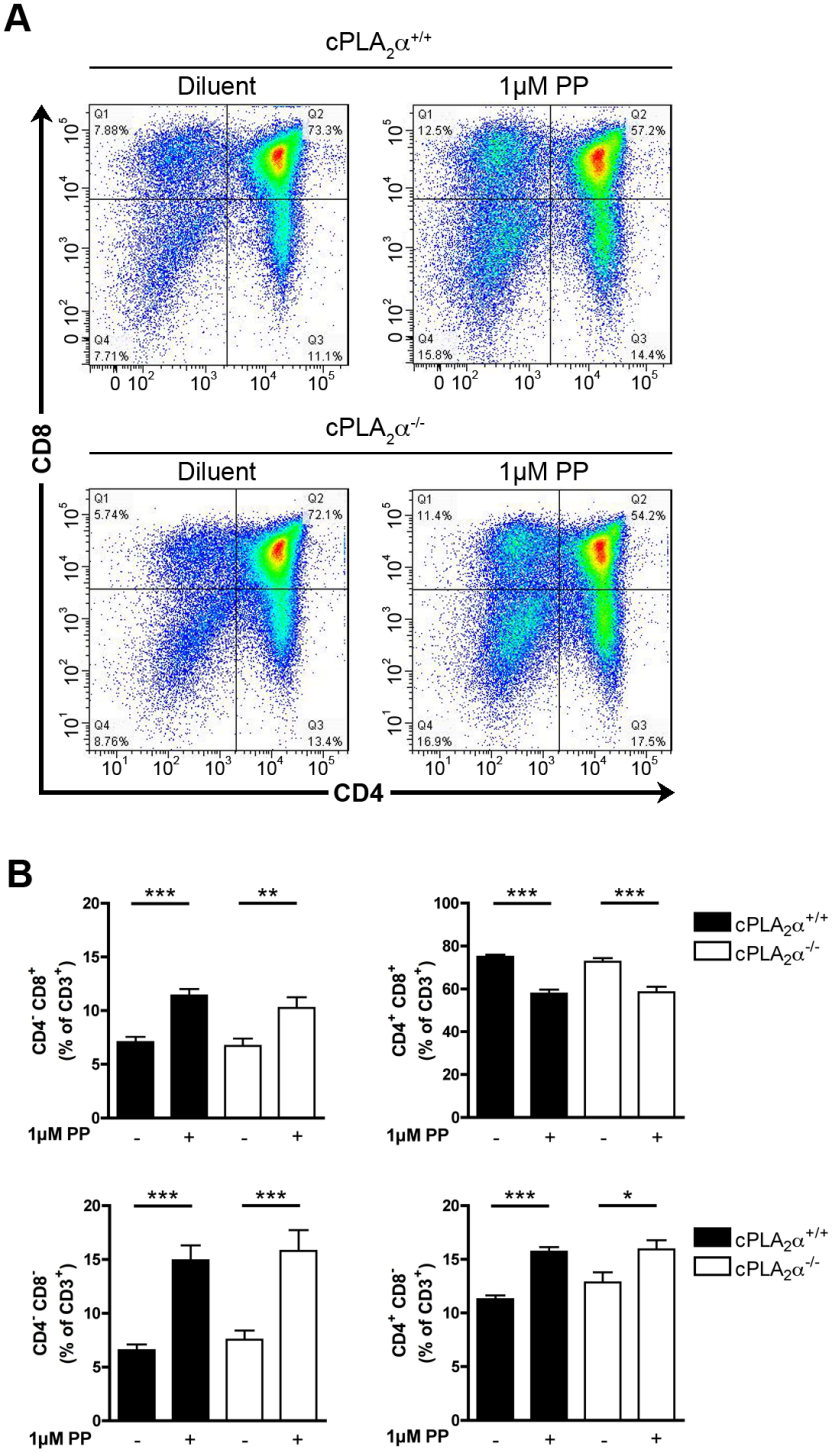
High concentration of PP impacts thymocyte maturation independently of cPLA_2_α inhibition. **A.** Representative distribution of the thymocyte subpopulations in WT and KO cPLA_2_α FTOCs after 5 days of culture in absence or presence of 1μM of PP. Thymocytes were identified with fluorochrome-conjugated antibodies directed against CD3, CD4 and CD8 and analyzed by flow cytometry. **B.** WT and KO cPLA_2_α fetal thymuses were cultured during 5 days as FTOCs in absence or presence of 1μM of PP. After mechanical dissociation of fetal thymuses, thymocytes were labeled with fluorochrome-conjugated antibodies directed against CD3, CD4, and CD8 and analyzed by flow cytometry. Data are mean ± SEM of 4 to 9 independent experiments and the number of fetal thymuses for each condition is: cPLA_2_^+/+^ and Diluent (n = 22); cPLA_2_^+/+^ and 1μM PP (n = 13); cPLA_2_^-/-^ and diluent (n = 9); cPLA_2_^-/-^ and 1μM PP (n = 6). * P< .05; ** P< .01; *** P< .001.

**Fig 6:**
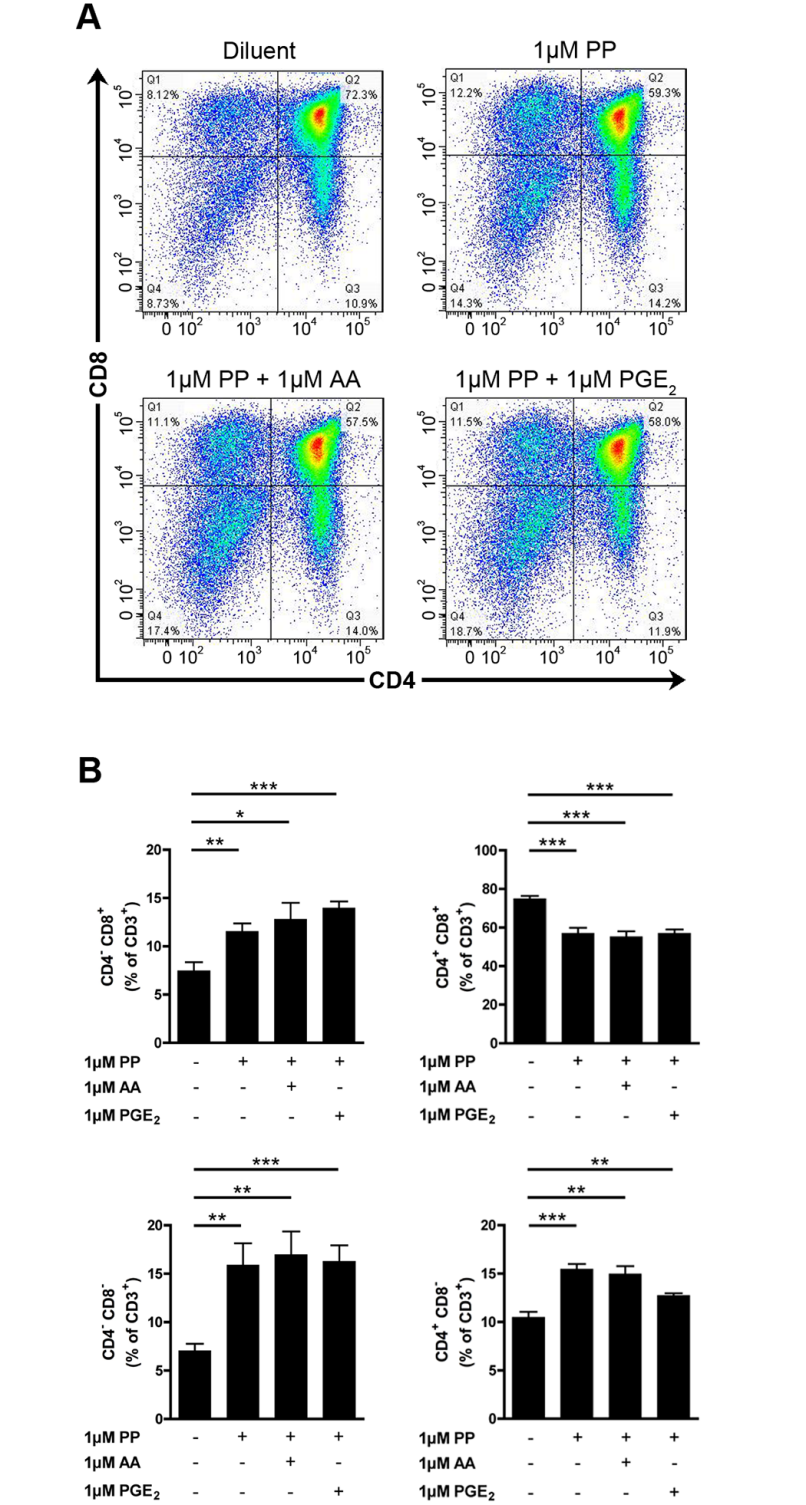
The unspecific effect of PP on thymocyte maturation is not reversed by exogenous AA and PGE_2_. **A.** Representative thymocyte subpopulation distribution in WT cPLA_2_α FTOC after 5 days of culture in absence or presence of 1μM of PP and exogenous (1μM) AA and PGE_2_. Thymocytes were identified cytofluorometrically using fluorochrome-conjugated antibodies directed against CD3, CD4 and CD8. **B.** WT cPLA_2_α fetal thymuses were cultured during 5 days as FTOCs in absence or presence of 1μM of PP, and exogenous (1μM) AA and PGE_2_. After mechanical dissociation of fetal thymuses, the thymocytes were identified with fluorochrome-conjugated antibodies directed against CD3, CD4, and CD8 and analyzed by flow cytometry. Data are mean ± SEM of 3 independent experiments and the number of fetal thymuses for each condition is: Diluent (n = 5); 1μM PP (n = 6); 1μM PP and 1μM AA (n = 5); 1μM PP and 1μM PGE_2_ (n = 5). * P< .05; ** P< .01; *** P< .001.

We next used a pharmacological approach to confirm our observations made in genetically engineered mice. The cPLA_2_α inhibitor pyrrophenone (PP) [48] suppresses AA release from an activated monocytic cell line and PGE_2_ release by renal mesangial cells with an IC_50_ of 24nM and 8nM, respectively [48]. WT FTOCs were cultured during 5 days in absence or in presence of 10 and 100 nm of PP and the repartitioned thymocyte subpopulations were analyzed by flow cytometry. As for the genetic approach, cPLA_2_α appeared dispensable, as no differences were observed in the maturation of thymocytes in presence of PP compared to those left untreated (Fig 3A and 3B). Rocca et al. and Xu et al. observed that the culture to FTOCs in presence of high concentrations (40μM) of the COX-2 inhibitor NS-398 led to the blockade of thymocyte differentiation [21, 35]. This effect of NS-398 was considered unspecific, as it was recapitulated in COX-2 deficient FTOCs and it was not reversed by the addition of PGE_2_ [35]. Using high concentrations (1μM) of the cPLA_2_α inhibitor, we observed an increase and a decrease of DN and DP thymocyte populations, respectively (Fig 4A and 4B). Furthermore, we observed an increase of the two SP populations (Fig 4A and 4B). Thus, high dose of PP affects the maturation of mouse thymocytes. We next wished to confirm the specificity of the inhibitor, here using cPLA_2_α KO FTOCs. We observed that PP, used at 1 μM, impedes the maturation of thymocytes deficient in cPLA_2_α (Fig 5A and 5B). Furthermore, the exogenous addition of AA and of PGE_2_, which was reported involved in thymocyte maturation [21], to PP-treated FTOCs did not restore normal thymocyte maturation (Fig 6A and 6B). Taken together, these results demonstrate that high doses of PP inhibit thymocyte maturation through the inhibition of another target than cPLA_2_α, most likely irrelevant to AA and prostaglandin release.

### cPLA_2_α gene disruption does not impact thymocyte maturation in the adult mouse

**Fig 7:**
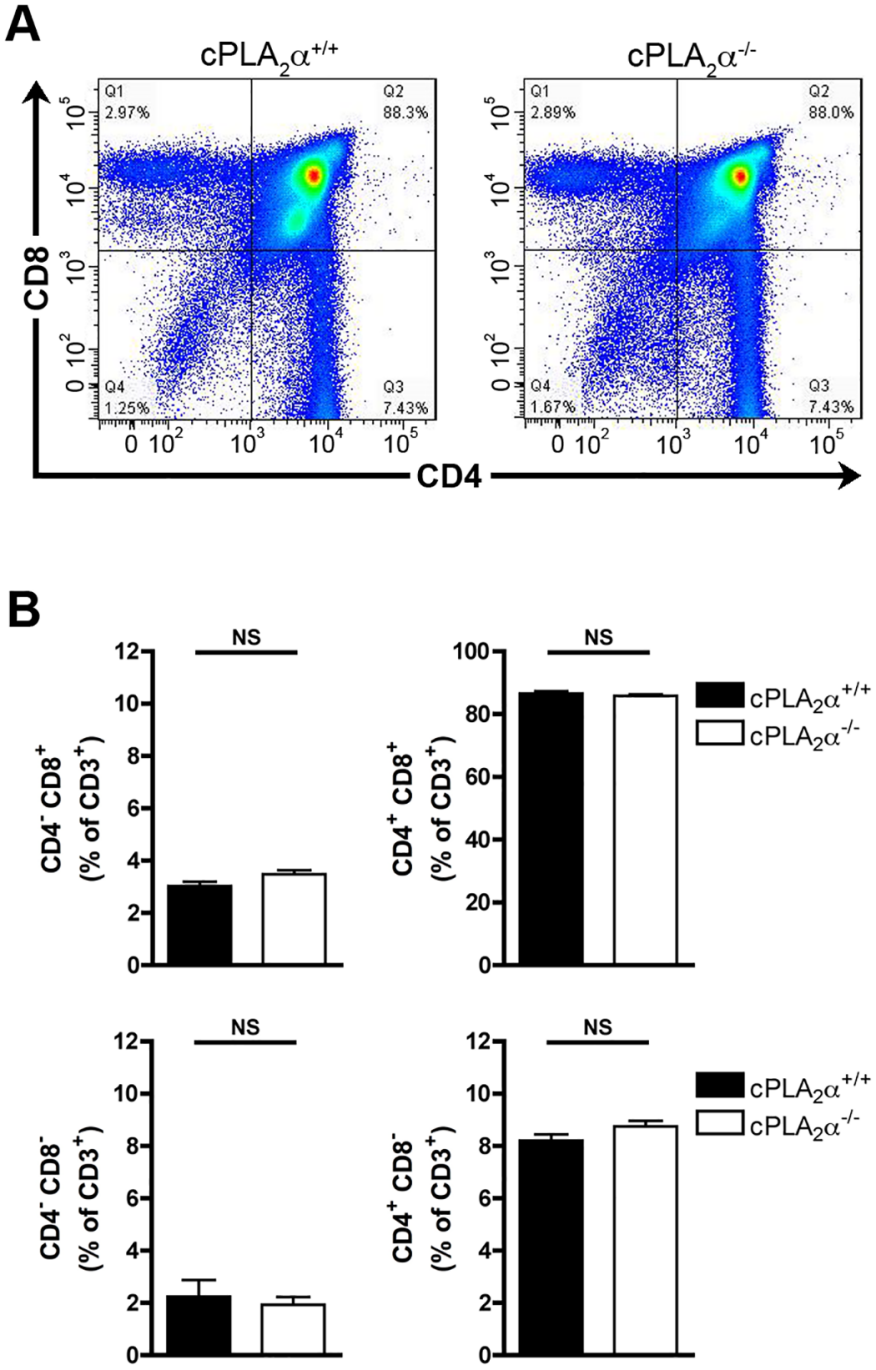
cPLA_2_α gene disruption does not affect thymocyte maturation in adult mice. **A.** Representative thymocyte subpopulation distribution in WT and KO cPLA_2_α adult mouse. Thymocytes were identified cytofluorometrically with fluorochrome-conjugated antibodies directed against CD3, CD4 and CD8. **B.** WT and KO cPLA_2_α adult mouse thymuses were dissociated mechanically and thymocytes were labeled with fluorochrome-conjugated antibodies directed against CD3, CD4, and CD8. Data are mean ± SEM of 7 independent experiments and the number of thymuses for each genotype is: cPLA_2_^+/+^ (n = 11); cPLA_2_^-/-^ (n = 11). NS (non significant).

Prior studies evaluated the role of prostaglandins in thymocyte maturation in the adult [21]. Having confirmed that cPLA_2_α is dispensable in thymocyte maturation at a fetal development stage in mice, we thus verified whether cPLA_2_α might be involved in thymocyte maturation in adult mice. The different thymocyte subsets were determined in cPLA_2_α WT and cPLA_2_α KO thymuses from adult (6–8 weeks) littermate mice. No differences in the proportions of the thymocyte subpopulations were observed between WT and KO thymuses (Fig 7A and 7B). Thus, the cPLA_2_α is dispensable for normal thymocyte maturation in adult mice.

### Pharmacological inhibition of cPLA_2_α does not impact human thymocyte maturation

Having demonstrated that the maturation of fetal and adult mouse thymocytes could proceed independently of cPLA_2_α, we wished to confirm our observations using human thymocytes. For this, we used thymuses from human newborns and young children suffering of cardiac malformation and undergoing thymectomies.

**Fig 8:**
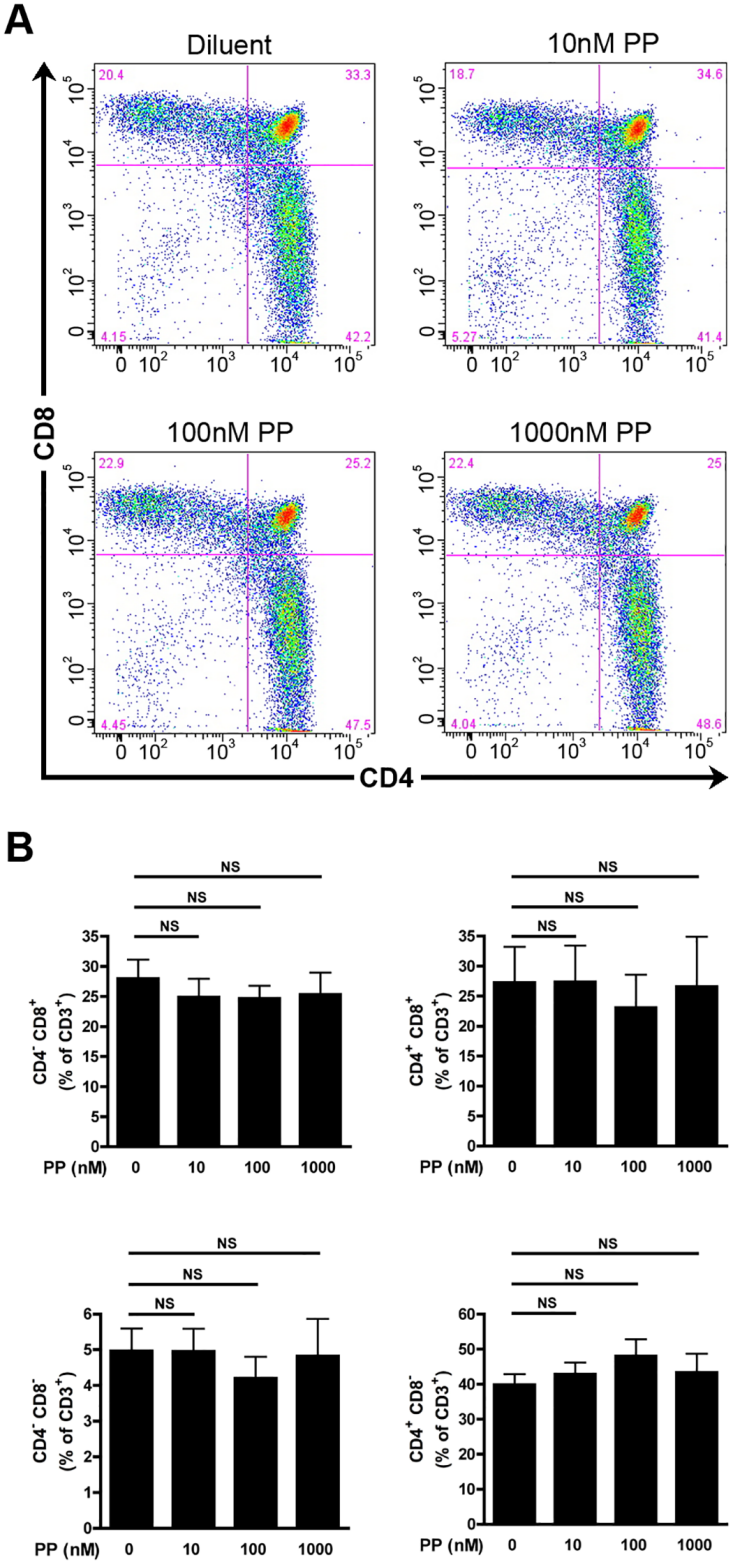
cPLA_2_α inhibition does not impact human thymocyte maturation. **A.** Representative thymocyte subpopulation distribution in human FTOC after 5 days of culture in absence or presence of indicated concentrations of PP. Thymocytes were identified cytofluorometrically using fluorochrome-conjugated antibodies directed against CD3, CD4 and CD8. **B.** Human FTOCs were cultured during 5 days in absence or presence of indicated concentrations of PP. After mechanical dissociation of human FTOCs, the thymocytes were labeled with fluorochrome-conjugated antibodies directed against CD3, CD4, and CD8 and analyzed by flow cytometry. Data are mean ± SEM of 3 independent experiments and the number of thymuses for each condition is: Diluent (n = 6); 10nM PP (n = 6); 100nM PP (n = 6); 1000nM PP (n = 6). NS (non significant).

To determine the role of the cPLA_2_α in human thymocyte maturation, small sections of human thymuses were cultured in absence or in presence of different concentrations of PP and then the thymocyte subpopulations were determined cytofluorometrically. We observed no differences in the percentage of different thymocyte subsets (DN, DP, SP CD4^+^ and SP CD8^+^) when thymuses were treated with PP up to 1μM (Fig 8A and 8B). Thus, cPLA_2_α appears dispensable for the maturation of human thymocytes.

## Discussion

In this study, we reveal for the first time the elaborated set of eicosanoids produced by the thymus through different stages of thymocyte development. LTB_4_ and LXA_4_ were the most abundant eicosanoids found in thymus. LTB_4_ is a recognized pro-inflammatory mediator involved in phagocyte chemotaxis, [49] while LXA_4_ displays anti-inflammatory activities and mediates clearance of apoptotic cells [50, 51]. LTB_4_ and LXA_4_ might play roles in the thymus, such as the recruitment of phagocytes and the stimulation of apoptotic cell clearance. The actual role of these lipids in the thymus is worth investigating, especially when it is considered that 98% of the thymocytes die by apoptosis in the thymus [52, 53]. Furthermore, we showed that the eicosanoid expression profile is modulated through the differen t thymocyte maturation stages, pointing to tight regulation of enzymes implicated in eicosanoid generation in the thymus. Future studies are thus necessary to verify the role of eicosanoids in thymus and the regulation mechanisms behind their production.

Through its important role in eicosanoid production, cPLA_2_α plays major roles in several physiological and pathophysiological processes, including immunity, reproduction, cancer and inflammation [7, 37–45]. Prostaglandins and their receptors are expressed in the thymus, and prior studies suggested that they are necessary for proper thymocyte maturation. Furthermore, it was demonstrated that the thymus is the organ with the highest concentration of thromboxane receptor, which is mostly expressed on DP thymocytes and appears implicated in the induction of thymocyte apoptosis [22] [25]. Herein, we surmised that cPLA_2_α might participate in eicosanoid generation and thymocyte maturation. To our surprise, we observed that production of most abundant eicosanoids and thymocyte maturation in the thymus occur independently of cPLA_2_α.

While cPLA_2_α is expressed in thymocytes [46] and its mRNA expression is modulated throughout development (S1 Fig), the exact role of cPLA_2_α in the thymus thus remains obscure. We investigated the impact of cPLA_2_α on the major populations of thymocytes based on surface expression of CD3, CD4 and CD8 receptors. However, cPLA_2_α and its products might have more subtle roles, and might regulate the development of other T-cell subpopulations such as T regs and γδ T-cells. Indeed, we showed that absence of cPLA_2_α has an impact on some less abundant eicosanoids. Whether these eicosanoids, and thus the cPLA_2_α, are involved in the function or development of scarce cellular populations is unknown. Furthermore, cPLA_2_α might be implicated in the production of eicosanoids that both positively and negatively regulate maturation of thymocytes. Thus, the overall effects of cPLA_2_α deficiency on thymocyte phenotype would be imperceptible. Finally, lysophosphatidic acid is involved in lymphocyte transmigration from the high endothelial venules of lymph nodes [54]. Whether cPLA_2_α and its products are also implicated in processes such as thymocyte entry or egress is unknown. As cPLA_2_α and AA metabolites are expressed in the thymus, the delineation of their exact role in the establishment of T-cell repertoire remains of great interest.

The prior demonstration of a role of prostaglandins in thymocyte maturation [21] was our impetus for our investigation of cPLA_2_α in the thymus. However, the actual role of prostaglandins in thymocyte maturation is currently debated. Indeed, two distinct studies reported divergent results. Whereas a first study suggested that COX-1 and COX-2-derived PGE_2_ participate in thymocyte maturation [21], a second one described that mice lacking expression of COX-1 and COX-2, EP-1 and EP-2 display normal thymocyte maturation [35]. What explains the discrepancies between these two studies is unclear, but we speculate that specific housing animal facility environment or background genetic drift might have contributed. Our results cannot settle the debate. Indeed, cPLA_2_α is not the only PLA_2_ enzyme expressed in the thymus [55–57] and other enzymes might participate to prostaglandin production in its absence. Hence, PGE_2_ levels are not altered by the absence of cPLA_2_α in the thymus (Fig 1B and 1C). Furthermore, sPLA_2_ X, which is highly efficiently at releasing AA from the cellular outer leaflet, is also expressed in the thymus [55, 56, 58, 59]. As we also excluded sPLA_2_ X in thymocyte maturation (S2A and S2B Fig), other PLA_2_ and or lipases expressed in thymus [56, 57] might thus compensate the absence of the cPLA_2_α and sPLA_2_ X for the production of prostaglandins,

We further observed that high concentrations of the cPLA_2_α inhibitor PP impair thymocyte maturation in mice, but not in humans. Similarly to the observations made by Xu et al. using high concentrations of the COX-2 inhibitor NS-398, [35] we demonstrated that the effect of PP at high concentrations (around 125 time higher than the IC_50_) is independent of its ability to inhibit its specific target. It seems unplausible that the unspecific target(s) of NS-398 and PP are the same. Indeed, the two compounds are structurally highly different and the unspecific effects observed on thymocytes are also distinct. Given that PP has no impact on human thymocyte development, we suggest that its unspecific target expressed in mice has no human ortholog, or that the human ortholog has a much lower affinity for the inhibitor. An unspecific effect of PP has recently been reported in a distinct study [60]. The authors demonstrated that the release of AA and lactate dehydrogenase from cPLA_2_α KO fibroblasts was efficiently inhibited by PP through the prevention of mitochondrial calcium uptake. The inhibition of this process in FTOCs could explain the reduction in thymocyte maturation but remains to be established.

In sum, our study provides novel information concerning the broad repertoire of eicosanoids present in the thymus and on the role of cPLA_2_α in thymocyte development. As a plethora of molecules drive T-cell functions in lymphoid organs and in the periphery, our study adds to the comprehension of mechanisms that are key in immunity.

## Supporting Information

S1 Fig cPLA_2_α mRNA expression is modulated according to the development stage. Relative expression of cPLA_2_α mRNA in mouse thymuses at E15.5, E18.5, 4–6 weeks, 6 months (and older) of age was determined by RT-QPCR and 2^-ΔCt^ methods. Data are mean ± SEM of 3 independent experiments.
(TIF)

S2 Fig sPLA_2_ X gene disruption does not affect thymocyte maturation in FTOC. **A.** Representative thymocyte subpopulation distribution in WT and KO sPLA_2_ X FTOC after 5 days of culture. Thymocytes were identified by flow cytometry using fluorochrome-conjugated antibodies directed against CD3, CD4 and CD8. **B.** WT and KO sPLA_2_ X fetal thymuses were cultured during 5 days as FTOCs. After mechanical dissociation of fetal thymuses, thymocytes were labeled with fluorochrome-conjugated antibodies directed against CD3, CD4, and CD8 and analyzed by flow cytometry. Data are mean ± SEM of 4 independent experiments and the number of fetal thymuses for each genotype is: sPLA_2_ X^+/+^ (n = 7); sPLA_2_ X^-/-^ (n = 13). NS (non significant).
(TIF)
