# Supplementary figures and images for: Study of the Role of Cytosolic Phospholipase A2 Alpha in Eicosanoid Generation and Thymocyte Maturation in the Thymus
Source: PLoS One. 2015 May 13;10(5):e0126204. doi: 10.1371/journal.pone.0126204 (PMC4430275; doi:10.1371/journal.pone.0126204)

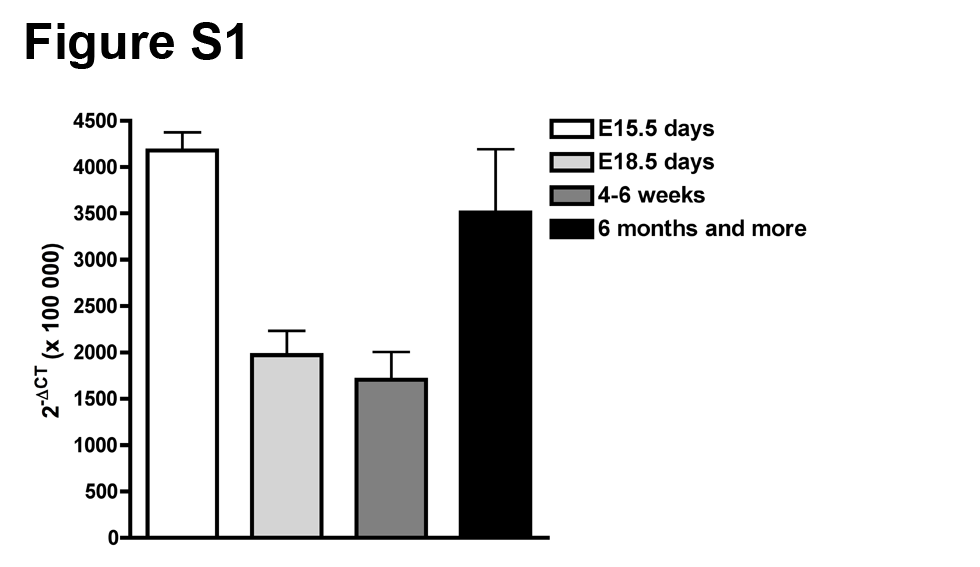

Supplement: S1 Fig — Relative expression of cPLA2α mRNA in mouse thymuses at E15.5, E18.5, 4–6 weeks, 6 months (and older) of age was determined by RT-QPCR and 2-ΔCt methods. Data are mean ± SEM of 3 independent experiments. (TIF) [file pone.0126204.s001.tif]

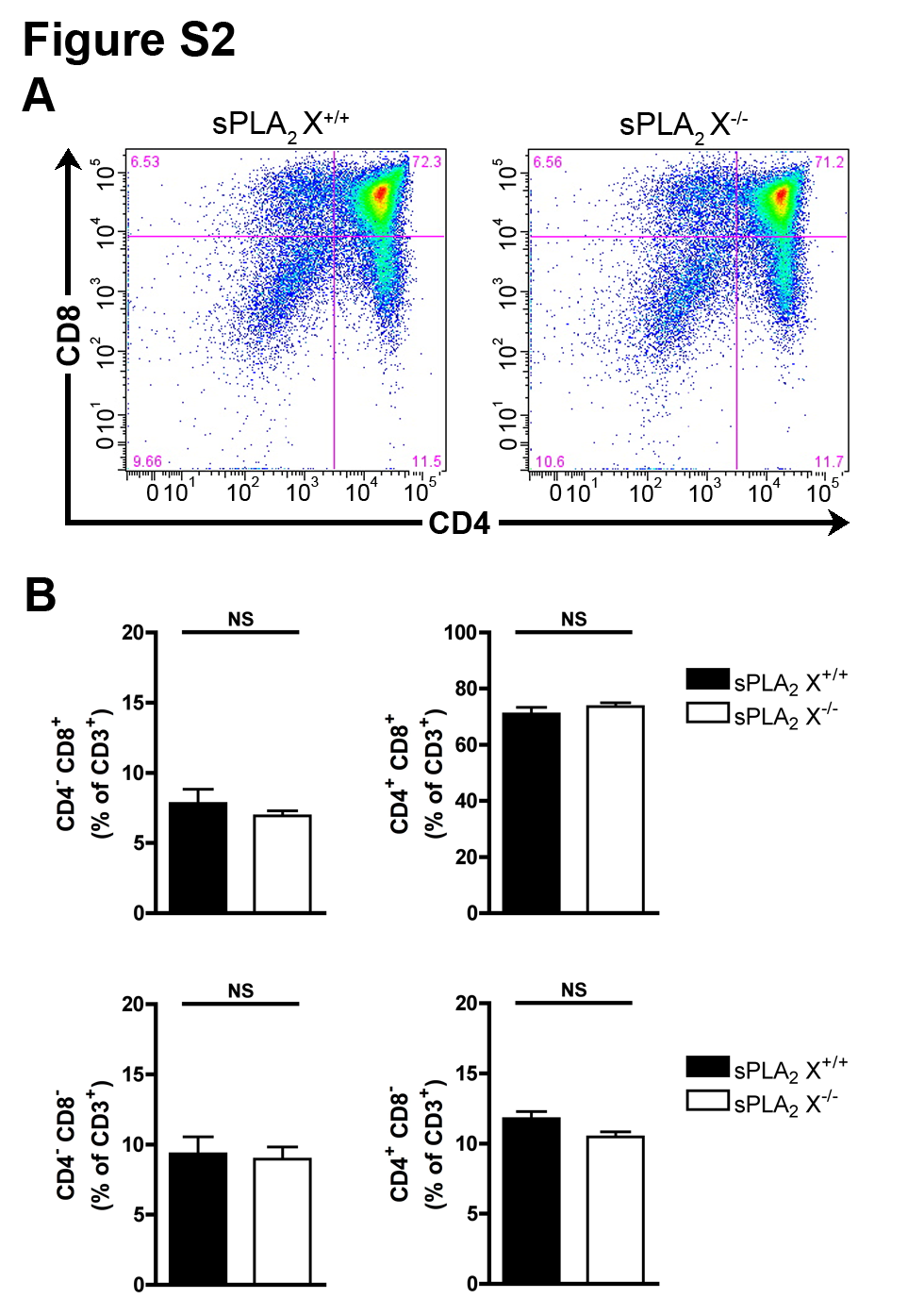

Supplement: S2 Fig — A. Representative thymocyte subpopulation distribution in WT and KO sPLA2 X FTOC after 5 days of culture. Thymocytes were identified by flow cytometry using fluorochrome-conjugated antibodies directed against CD3, CD4 and CD8. B. WT and KO sPLA2 X fetal thymuses were cultured during 5 days as FTOCs. After mechanical dissociation of fetal thymuses, thymocytes were labeled with fluorochrome-conjugated antibodies directed against CD3, CD4, and CD8 and analyzed by flow cytometry. Data are mean ± SEM of 4 independent experiments and the number of fetal thymuses for each genotype is: sPLA2 X+/+ (n = 7); sPLA2 X-/- (n = 13). NS (non significant). (TIF) [file pone.0126204.s002.tif]
